# Cross-cultural adaptation and psychometric properties of the Dutch version of the Hand Function Sort in patients with complaints of hand and/or wrist

**DOI:** 10.1186/s12891-019-2649-2

**Published:** 2019-06-07

**Authors:** Annemiek Muskee, Redmar J. Berduszek, Rienk Dekker, Michiel F. Reneman, Corry K. van der Sluis

**Affiliations:** University of Groningen, University Medical Center Groningen, Department of Rehabilitation Medicine, Hanzeplein 1, Groningen, 9713 GZ The Netherlands

**Keywords:** Cross-cultural comparison, Musculoskeletal diseases, Arm, Surveys and questionnaires, Psychometrics

## Abstract

**Background:**

Musculoskeletal complaints of arm, neck, and shoulder (CANS) can lead to loss of work productivity. To assess the functional consequences of impairments in work, patient-reported outcomes can be important. The Hand Function Sort (HFS) is a 62-item pictorial questionnaire that focuses on work task performance. The aims of this study were the cross-cultural adaptation of HFS into HFS-Dutch Language Version (HFS-DLV) (Part I) and determining construct validity, internal consistency, test-retest reliability, responsiveness and floor/ceiling effects of HFS-DLV (Part II).

**Methods:**

I: Translation into Dutch using international guidelines. II: Construct validity was assessed with Spearman’s correlation coefficients between the HFS-DLV and the Dutch version of the QuickDASH, PRWHE, PDI, RAND-36, NRS-pain, and work ability score. Internal consistency was assessed using Cronbach’s α and reliability by a test-retest procedure. A global rating scale of change was used after 4–8 weeks of hand therapy to determine responsiveness.

**Results:**

I: Forty patients were included, and no items were changed. II: 126 patients with hand, wrist, and/or forearm disorders classified as specific or nonspecific CANS. Six predefined hypotheses (50%) were confirmed. Cronbach’s α: 0.98. Test-retest reliability: ICC of 0.922. AUC of 0.752. There were no floor/ceiling effects.

**Conclusions:**

I: Translation process into the HFS-DLV went according to plan. II: For construct validity, the presumed direction of correlations was correct, but less than 75% of hypotheses were confirmed. Internal consistency was high, suggesting redundancy. Reliability and responsiveness of the HFS-DLV were good. HFS-DLV can be used in research or clinical practice for Dutch patients with CANS, to evaluate self-reported functional work ability.

## Background

Musculoskeletal complaints of arm, neck, and shoulder (CANS) not caused by acute trauma or systemic disease can lead to considerable disability [1–4] and a substantial loss of productivity at work [5]. A broad range of 12-month prevalence of CANS can be found, from 2.3–41% [6]. In the working population, a 12-month prevalence of 22–40% was reported [7].

To assess work abilities and to help interpret the functional consequences of impairments in work, patient-reported outcomes (PROs) can be important [8]. In rehabilitation medicine, PROs provide insights to guide decision-making in interventions and evaluate treatment effects [9, 10]. Knowledge of a self-reported perception of ability can be an important indicator of functional status [10].

PROs can be classified into different categories, including generic, disease-specific, or region-specific (i.e., focusing on a specific region, such as the upper extremity) [9, 11]. A region-specific measure can be used for patients with different disorders and is therefore more practical in daily use [11]. PROs are usually short questionnaires that can be administered before or after a clinical evaluation. Most PRO questionnaires are developed in English [12] and should be translated and adapted to different languages and cultures because there can be relevant differences in disease terminology and general cultural differences [12, 13]. Different PROs for complaints of the upper extremities are available, including the Patient-Rated Wrist/Hand Evaluation (PRWHE), the Disabilities of the Arm, Shoulder, and Hand outcome measure (DASH), and its shortened version, the QuickDASH [14–16]. These PROs focus on upper extremity function in daily life and symptoms, including pain. They include items that are related to the functional ability to work but do not address this directly.

The 62-item Hand Function Sort (HFS) was developed to quantify the physical ability to work and perform daily life activities [10]. The HFS is a self-reported, region-specific questionnaire that represents tasks across a range of physical demands and focuses on upper extremity performance in work tasks and other activities of daily living [10]. The HFS can be used for the quantification of work disability and for determining the ability to perform a particular job and its outcome can be used to guide Functional Capacity Evaluation [10]. The HFS can be used for patients with CANS, as it has been shown that these complaints are frequently work-related [2, 4, 5]. The developers of the HFS found that the perception of functional ability can be a predictor of a return to work [8]. Since the HFS is pictorial, it can be used with a broad range of patients, including low literacy patients, an advantage most PROs do not have.

Before translated PROs can be used, a proper validation of the measurement instrument is necessary [17]. The HFS has been validated in English using construct validation in two approaches [10], and recently the HFS was translated and validated into French [18]. The HFS has not yet been translated into Dutch. Therefore, the first aim of this study was the cross-cultural adaptation of the HFS into the HFS-Dutch Language Version (HFS-DLV). The second aim was to determine the psychometric properties of the HFS-DLV, including construct validity, internal consistency, test-retest reliability, responsiveness, and floor/ceiling effects.

## Methods

### Part 1: cross-cultural adaptation of the HFS-DLV

For the translation of the HFS, the guidelines of Beaton were followed [13]. Two native Dutch translators each wrote a translation from English into Dutch (T1 & T2). One of the translators was aware of the concepts being studied (informed), the other translator was not (uninformed). They both produced a written report, including comments and the rationale for their choices. These translations were synthesized into T-12 by the two translators and an observer, whereby consensus was reached on discrepancies. Two native English translators, who spoke Dutch fluently, made two back translations (BT1 & BT2) of the T-12 version into English. They were uninformed about the concepts of the study and had no medical background. An expert committee, consisting of two specialists in rehabilitation medicine (RJB & CKS), a methodologist, and the translators (forward and back translators), reviewed all the versions, and consensus was reached on discrepancies. This resulted in a prefinal version of the HFS-DLV. A total of 30–40 patients was recommended for testing this prefinal version [13]. Participants were included from the outpatient clinic of the department of rehabilitation medicine of a university hospital. All participants were receiving hand therapy and were asked to complete the prefinal version of the HFS-DLV after their therapy appointment. Inclusion criteria were: age 18 years or over and specific or nonspecific complaints of the hand, wrist and/or forearm [1]. Patients with complaints caused by trauma were included, but only if the trauma was more than 3 months ago. Patients with complaints of stable osteoarthritis were also included. Patients with insufficient knowledge of the Dutch language or with other medical conditions causing considerable disability in functioning (e.g. neurological disorders or joint disease) were excluded. In the presence of a researcher (AM) the participants completed the prefinal version and gave comments on the comprehensibility of the items. These comments were reviewed by two specialists in rehabilitation medicine (RJB & CKS), a methodologist and a researcher (AM). In this consensus meeting the HFS-DLV was finalized. During the translation process contact with the original developers of the HFS was maintained.

### Part 2: measurement properties of the HFS-DLV

#### Participants

Participants were included from the outpatient clinic of the department of rehabilitation medicine of a university hospital and from five locations of peripheral hand therapy practices in the northern part of the Netherlands. Inclusion criteria were: age 18 years or over and specific or nonspecific complaints of the hand, wrist, and/or forearm [1]. CANS was defined as musculoskeletal complaints of arm, neck, and shoulder not caused by acute trauma or systemic disease [1]. We only included patients with complaints of the hand, wrist, and/or forearm, as we expected the most direct effects of these specific complaints on the hand function, as measured by the HFS. Exclusion criteria were identical to part 1.

#### Procedure

In this prospective observational study, participants completed the HFS-DLV and the Dutch version of the QuickDASH, PRWHE, Pain Disability Index (PDI), RAND-36, Numeric Pain Rating Scale (NRS-pain), and Work Ability Score (WAS). Measurement properties were assessed using the definitions of the COSMIN group [19].

If participants were included in the university hospital, the questionnaires were sent by mail. When included in a peripheral hand therapy practice, the participants had the option to complete the questionnaires directly after their therapy appointment or to complete the questionnaires at home and return to the researcher by mail. The second set of questionnaires was sent and returned by mail.

#### Questionnaires

We used the Dutch validated versions of all questionnaires, which were available for free. For the use of the Hand Function Sort, we had permission from the developer. The HFS-DLV is a 62-item pictorial questionnaire, wherein each item consists of a drawing of a task accompanied by a task description. Answers are given on a 5-point scale from *able* to *unable* (a “?” option is present for “I don’t know”). An overall rating of perceived capacity (RPC) score can be calculated with ranges from 0 to 248, where a higher score indicates a better perceived capacity.

The HFS includes an internal reliability check: first, by checking three pairs of highly similar items for consistency (≥4 points difference between the similar items indicates an unreliable test) and second, by counting the total number of “?” answers (if ≥6 “?” answers are filled in, the test is marginally reliable). A questionnaire cannot be qualified as unreliable based on only too many “?”, the difference between similar items should also be taken into account. Marginally reliable questionnaires will be included in the analysis; unreliable questionnaires will be excluded from the analysis.

All the items in the HFS are assigned to a five-level physical demand characteristics (PDC) system. This system can be used to categorize the demands of a given work position [8, 10]. Items 1–16 of the HFS correspond to sedentary activities, items 17–34 to light activities, items 25–52 to medium activities and items 53–62 to heavy activities. An RPC score for each PDC level can be calculated. Minimum total RPC scores that would be necessary to function at a specific PDC level have been proposed: sedentary (100–136), light (154–190), medium (200–228), heavy (238–248), and very heavy. In this way, the HFS can be used to indicate a person’s perception of capacity for different work demands [8].

The QuickDASH is an 11-item questionnaire that measures symptoms and physical function involving disorders of the upper limb. It has a summative score on a 100-point scale, where a score of 100 indicates the most disability [14]. It has been shown to have good reliability, validity, and responsiveness in English [14, 20]. Previous research shows that the QuickDASH performs comparably to the DASH [14, 20, 21], but is preferable for conditions with functional limitations [22]. The DASH and QuickDASH have been translated into Dutch, and the DASH-Dutch Language Version has been validated [23].

The Patient Rated Wrist Evaluation (PRWE) [16], was modified into the PRWHE (H: Hand) [24]. It is a 15-item questionnaire designed to measure two modalities: wrist pain and disability (5 vs. 10 items). Both modalities are equally weighted, and the highest score is 100 (indicating the most pain and disability). The test-retest reliability is excellent, and validity and responsiveness are good [16, 24].

The PDI measures the extent to which chronic pain interferes with various life activities. An overall disability score is calculated by adding the scores of 7 items (categories of life activities), and ranges from 0 to 70 (a higher score indicates more disability) [25]. The PDI is a valid measure for pain-related disability, with a modest to good test-retest reliability [26, 27].

The RAND-36 is a health-related quality of life survey that consists of 36 items that assess eight health concepts: physical functioning, social functioning, role limitations (physical problem), role limitations (emotional problem), mental health, vitality, pain, and general health perception [28]. The internal consistency of the RAND-36 is high and the construct validity satisfactory [29]. Most subscales appear to be strong, unidimensional, and reliable, except for the subscales general health perception and vitality. Therefore, the latter subscales have a lower reliability. Scores are calculated on a 100-point scale, where a higher score indicates a better quality of life [29, 30].

The NRS-pain scale is a 11-point scale measuring pain intensity, ranging from 0 (no pain) to 10 (worst imaginable pain) [31].

The WAS is a single-item instrument, which measures the current work ability in relation to lifetime best [32].

#### Construct validity

Construct validity is the degree to which the scores of the measurement are consistent with hypotheses [33]. Validity was determined by assessing construct validity because no gold standard was available. To determine construct validity, a total of 50 participants is required [33].

Construct validity was assessed using correlation coefficients to determine the relationship between the HFS-DLV and the Dutch version of the QuickDASH, PRWHE, PDI, RAND-36, NRS-pain, and WAS. The HFS-DLV focuses on upper extremity work task performance and disability; we therefore assumed a strong correlation of the HFS-DLV with the QuickDASH and PRWHE. With the PDI, RAND-36 (physical functioning), and the WAS, a moderate-strong correlation was assumed as these questionnaires assess (dis)ability in a similar matter as the HFS, but they do not focus on the upper extremities. Because the HFS does not focus on mental health and pain in particular, we assumed a weaker correlation with specific concepts of the RAND-36 and the NRS-pain. Nine predefined hypotheses about the assumed correlation with other questionnaires were proposed (Table 1).

**Table 1:** Assumed correlations of the HFS-DLV with other questionnaires

Correlation^a^	Questionnaires
Strong-very strong	QuickDASH, PRWHE
Moderate-strong	PDI, RAND-36 (physical functioning), WAS
Weak-moderate	RAND-36 (social functioning, vitality), NRS-pain
Weak	RAND-36 (mental health)

^a^0.00–0.25: weak; 0.26–0.50: moderate; 0.51–0.75: strong; above 0.75: very strong [34]

Furthermore, three predefined hypotheses for known groups validity were proposed, determined by a Mann-Whitney U test. Some of the tasks in the HFS-DLV have a higher PDC level and require strength, therefore, we assumed from a biological perspective that males would be able to do these tasks in an easier fashion and have a higher overall score as a result [35]. Second, it has been shown that younger age, better perceived general health, and higher beliefs of pain self-efficacy are associated with higher work ability and the continuance of work in patients with chronic nonspecific musculoskeletal pain [36]. Therefore, we assumed that the employed population would experience less disability in work task performance and would score higher on the HFS as compared to unemployed persons. Third, it was proposed that when the dominant hand is affected, this will result, at least for some upper extremity conditions, in more functional disability [37]. Thus, we assumed a lower score on the HFS-DLV when the dominant side was affected, as also has been shown for the English HFS [10] and the QuickDASH [38]. The HFS-DLV was considered valid when 75% of the hypotheses were met.

#### Internal consistency

Internal consistency is the degree of the interrelatedness among the items and was determined using Cronbach’s α, where a value between 0.70 and 0.90 was considered acceptable [33]. To determine the internal consistency, a total of 434 participants is recommended by the COSMIN group (7 times the number of items; i.e. 7 × 62 items) [33].

#### Test-retest reliability

Reliability is the degree to which the measurement is free from measurement error. To assess test-retest reliability a total of 50 participants is recommended [33]. Consecutive participants included in the university hospital were asked to complete the HFS after 1–3 weeks for a second time, until the desired number of 50 participants was reached. This interval was assumed long enough to prevent recall and allow administration of questionnaires by mail, yet short enough to ensure no clinical change occurred. A test-retest procedure was used to calculate the intraclass correlation coefficient (ICC) for agreement (two-way mixed effects model) and limits of agreement (LoA) using the Bland-Altman method [39]. ICC was considered acceptable above 0.70 and good above 0.80 [33].

#### Responsiveness

Responsiveness is the ability to detect change over time in the construct to be measured. To assess responsiveness, a total of 50 participants is recommended [33]. Consecutive participants included in the peripheral hand therapy practices were asked to complete the questionnaire for a second time after 4–8 weeks of hand therapy provided by a certified hand therapist, until the desired number of 50 participants was reached. A criterion approach (anchor-based method) was used with a global rating scale (GRS) as a gold standard. At follow-up, participants were asked a question to indicate their overall perceived change on a 7-point scale, ranging from 1 (much better) to 7 (much worse). For the analysis, a score of 1 or 2 was considered an improvement, a score of 3, 4, or 5 was considered stable, and a score of 6 or 7 was considered as a decline in complaints [40]. The area under the ROC curve (AUC) was assessed, and an AUC of at least 0.70 was considered appropriate [33]; a minimal important change (MIC) was determined by a ROC cut-off point associated with optimal sensitivity and specificity [41]. The standard error of measurement (SEM) was calculated by performing an ANOVA and taking the square root of the within groups mean square. The SEM was used to calculate the smallest detectable change (SDC) using the formula SDC = 1.96 × √2 × SEM. The SDC should be smaller than the MIC [33].

#### Floor and ceiling effects

Floor and ceiling effects can occur when a high proportion of the total population has a score at the lower or upper end of the scale [33]. These were considered to be present if more than 15% of participants reached the maximum or minimum score [33].

#### Statistical analysis

For the statistical analysis, SPSS (IBM SPSS Statistics for Windows 2013 v22.0, Armonk, NY: IBM Corp) was used. A *p* <  0.05 was considered to be of statistical significance. The distribution of the data was assessed by graphical methods (Q-Q plot) to determine the use of parametric or nonparametric tests.

## Results

### Part 1: cross-cultural adaptation of the HFS-DLV

During the translation process, problems with translating specific words emerged. The questionnaire was named HFS-DLV, since an adequate translation for HFS was not available. The main difficulty was finding the proper Dutch names for the tools and implements used (for example, *T-handle wrench*). Weights and distances had to be adjusted from imperial to metric system units (e.g., kilograms instead of pounds). Consensus for the T-12 was reached easily. The expert committee thoroughly examined and debated all the items before completing the prefinal version. A total of 40 participants completed the prefinal version of the HFS-DLV between April and August 2015 (Table 2). During administration of the prefinal version, comments for 35 items were registered. Most concerned the activity itself and not the language used. Item 54 “dig a hole for a fence post with a post-hole digger”, was commented on the most. For this activity, a different tool is used in the Netherlands; however, this tool does not resemble the instrument in the drawing. General comments included the items being too masculine (6 times) and that it was unclear which hand to use (11 times). Participants found that the pictures contributed to an understanding of the items. After discussion, we did not change any of the items nor the pictures, mainly because the alternatives provided by participants were not considered better and had already been discussed in the consensus meeting in which the prefinal version was completed.

**Table 2:** Participant characteristics of part 1: cross-cultural adaptation of the HFS-DLV and part 2: measurement properties

	Part 1 (*n* = 40)	Part 2 (*n* = 126)		
		Total (*n* = 126)	UH (*n* = 57)	PHTP (*n* = 69)
Gender, n (%)				
Male	20 (50%)	46 (37%)	20 (35%)	26 (38%)
Female	20 (50%)	80 (63%)	37 (65%)	43 (62%)
Age, median (IQR)	53 years (41–63)	48 years (32–60)	47 years (32–55)	52 years (35–66)
Diagnosis, n (%)				
Specific CANS	9 (23%)	69 (55%)	21 (37%)	48 (70%)
Nonspecific CANS	6 (15%)	57 (45%)	36 (63%)	21 (30%)
Posttraumatic complaints	19 (48%)	N/A	N/A	N/A
Osteoarthritis	6 (15%)	N/A	N/A	N/A
Dominant side affected, n (%)	26 (65%)	104 (83%)	48 (84%)	56 (81%)
Employed, n (%)	23 (58%)	73 (58%)	37 (65%)	36 (52%)
Questionnaire scores, median (IQR)				
HFS-DLV		145 (92–198)	151 (110–198)	141 (86–198)
QuickDASH		34 (20–50)	32 (19–50)	34 (20–51)
PRWHE		49 (25–68)	47 (23–67)	50 (29–72)

*UH* university hospital, *PHTP* peripheral hand therapy practices, *n* absolute number, *IQR* interquartile range, *CANS* Complaints of Arm, Neck, and Shoulder, *N/A* Not applicable, *HFS-DLV* Hand Function Sort-Dutch Language Version, *QuickDASH* Quick Disabilities of the Arm, Shoulder, and Hand Outcome Measure, *PRWHE* Patient Rated Wrist/Hand Evaluation

### Part 2: measurement properties of the HFS-DLV

#### Participants

The HFS was administered to 126 patients between December 2015 and August 2018 (Table 2). Patients included from the university hospital and peripheral hand therapy practices are shown separately. These two samples are similar based on gender, age, employment status and affected side. The diagnosis did differ between these samples (more nonspecific CANS in university hospital and more specific CANS in peripheral hand therapy practices).

**Fig. 1:**
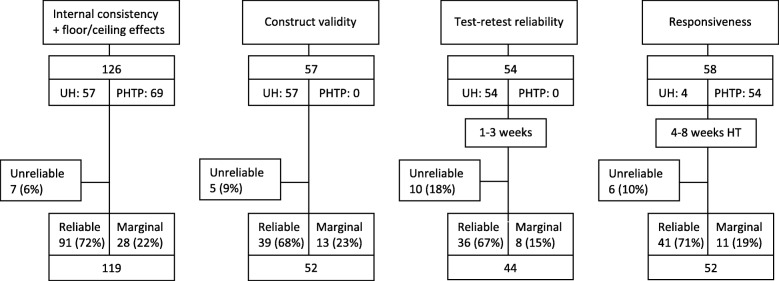
Flowchart inclusion procedure. UH: university hospital. PHTP: peripheral hand therapy practices. HT: hand therapy

Figure 1 shows the inclusion procedure for the different measurement properties and the total HFS-DLV questionnaires included. The internal reliability check of the HFS-DLV was used for determining if a questionnaire was reliable, marginal or unreliable (see Methods). Questionnaires completed by participants included for internal consistency (*n* = 119) were also used for construct validity (*n* = 52), test-retest reliability (*n* = 44), and responsiveness (*n* = 52).

#### Construct validity

In total, 6 out of 12 (50%) predefined hypotheses were accepted (Table 3). The predefined hypotheses for the correlations between HFS-DLV and NRS pain, RAND-36 vitality, and RAND-36 mental health were not accepted. For all three, a slightly higher correlation then predicted was found. Spearman’s correlation coefficient was used since the HFS-DLV and most of the other six questionnaires were not normally distributed.

**Table 3:** Spearman’s correlation coefficient *r*_*s*_ for construct validity and known groups validity (*n* = 52)

	Spearman’s correlation coefficient *r*_*s*_ HFS-DLV*	95% CI	*P* value	Predefined hypothesis	Hypothesis accepted
QuickDASH	−0.73	− 0.57 to − 0.84	< 0.001	0.51–1.00 (strong-very strong)	yes
PRWHE	− 0.62	− 0.42 to − 0.77	< 0.001	0.51–1.00 (strong-very strong)	yes
PDI	− 0.68	− 0.50 to − 0.80	< 0.001	0.26–0.75 (moderate-strong)	yes
WAS	0.61	0.40 to 0.76	< 0.001	0.26–0.75 (moderate-strong)	yes
RAND-36 physical functioning	0.58	0.37 to 0.74	< 0.001	0.26–0.75 (moderate-strong)	yes
RAND-36 vitality	0.57	0.35 to 0.73	< 0.001	0.00–0.50 (weak-moderate)	no
NRS pain	−0.52	−0.29 to − 0.70	< 0.001	0.00–0.50 (weak-moderate)	no
RAND-36 social functioning	0.44	0.19 to 0.64	0.001	0.00–0.50 (weak-moderate)	yes
RAND-36 mental health	0.43	0.17 to 0.63	0.002	0.00–0.25 (weak)	no
HFS-DLV: median score (IQR)			*P* value	Predefined hypothesis	
*Male (n = 19)*	*Female (n = 33)*				
182 (110–207)	151 (111–195)		0.38	male > female	no
*Employed (n = 35)*	*Unemployed (n = 17)*				
171 (136–200)	129 (63–185)		0.077	employed > unemployed	no
*Non-dominant affected (n = 9)*	*Dominant side affected (n = 43)*				
166 (109–206)	151 (126–208)		0.521	non-dominant side affected > dominant side affected	no

*95% CI* 95% confidence interval, *HFS-DLV* Hand Function Sort-Dutch Language Version, *QuickDASH* Quick Disabilities of the Arm, Shoulder, and Hand Outcome Measure, *PRWHE* Patient Rated Wrist/Hand Evaluation, *PDI* Pain Disability Index, *WAS* Work Ability Score, *NRS pain* Numeric Pain Rating Scale, *IQR* interquartile range

The three predefined hypotheses for known groups validity were not accepted because differences were not statistically significant. The median scores of the HFS-DLV were higher in the predicted groups, so there was a trend in the right direction (Table 3).

#### Internal consistency

Cronbach’s α for internal consistency was 0.98 (*n* = 119).

#### Test-retest reliability

**Fig. 2:**
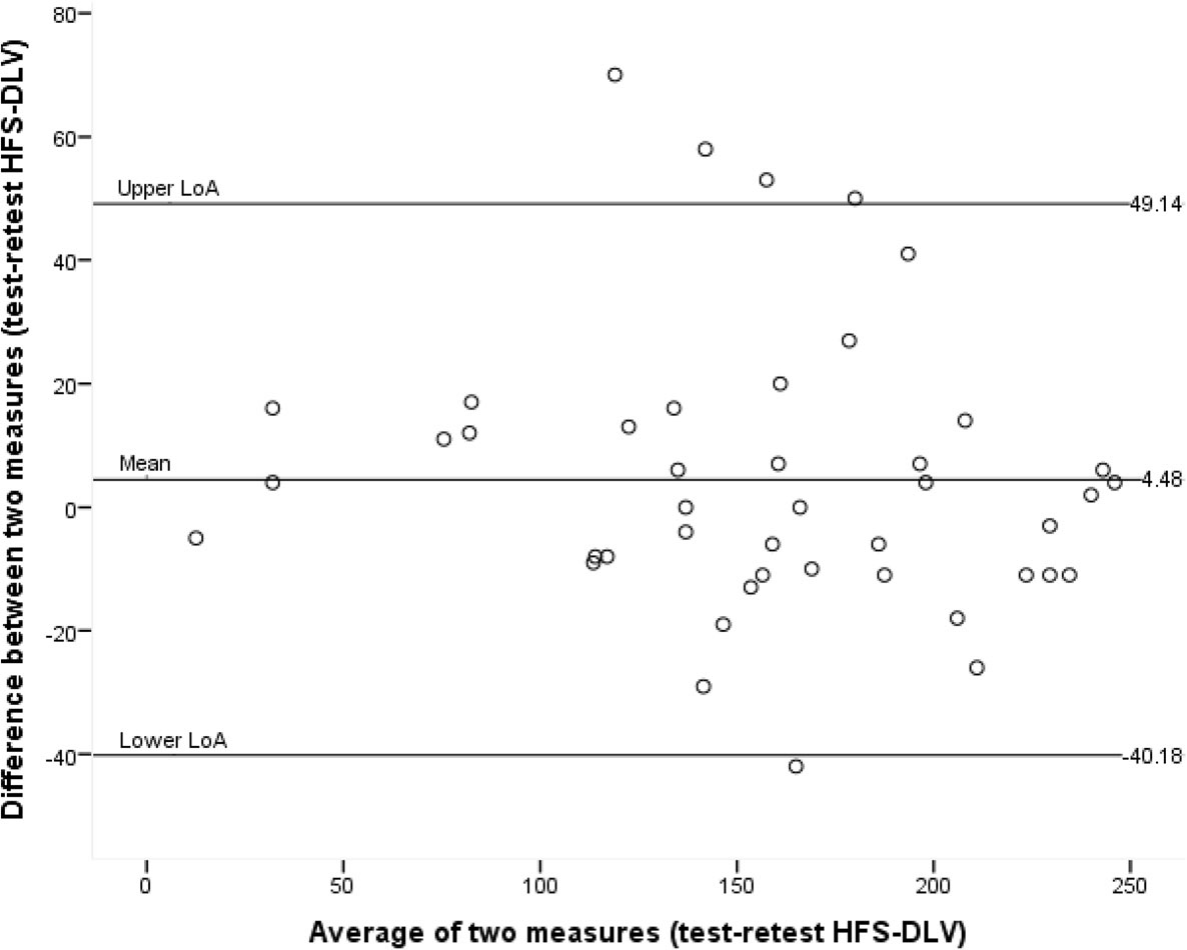
Bland-Altman plot. The middle line represents the mean difference between the test and retest of the HFS-DLV. The upper and lower lines represent the limits of agreement. HFS-DLV: Hand Function Sort-Dutch Language Version. LoA: limits of agreement

The median interval between the two completed questionnaires was 15 days (IQR 13–19). The ICC for test-retest reliability (*n* = 44) was 0.922 (95% CI: 0.861–0.956). The T-test of the difference between the first and second measurement of the HFS-DLV was not significant (*p* = 0.199). Using the Bland-Altman method, the mean difference between test and retest was 4.48 with 95% upper and lower limits of agreement of − 40.18 and 49.14 (Fig. 2).

#### Responsiveness

The median interval between the two completed questionnaires was 41 days (IQR 35–56). The AUC was 0.752 (*n* = 52), with a ROC cut-off point and MIC of 37/248 (sensitivity 0.619, specificity 0.903). The SEM was 16.2 and the SDC was 45/248.

#### Floor and ceiling effects

No participants (0%) had the lowest possible score, and only one participant (1%) had the highest possible score of 248. No floor or ceiling effects were found.

## Discussion

The cross-cultural translation and adaptation of the Hand Function Sort for Dutch-speaking patients was successfully performed in a thorough manner. As such, the HFS-DLV can be used for research purposes and in clinical practice. The psychometric properties of the HFS-DLS appeared to be good, although the construct validity needs further study.

### Part 1: cross-cultural adaptation of the HFS-DLV

A careful procedure, such as the 5-step translation and adaptation process as applied in this study, should be followed. In testing the prefinal version of the HFS-DLV, 98% of the participants made comments about the items and the comprehensibility in general. In contrast, Konzelmann et al. [18] stated that only 32% of participants made comments about the prefinal version of the French HFS. Having a researcher present in our setting might explain this difference. Therefore, for future translations of questionnaires, the presence of a researcher orally receiving comments should be considered.

Participants frequently commented that it was unclear which hand to use for the described tasks. The developers of the HFS were consulted regarding this comment. They explained that the self-selection of the participants to either demonstrate their inability to perform the task with the injured hand or their ability to perform the task with their residual capacity is an important psychological variable. This cannot be identified if the participants were instructed which hand to use. Thus, allowing the participants to self-select gives the researchers the opportunity to consider whether and to what degree the participants may be magnifying their symptoms. We recommend adding an explanation to the examiner’s manual about this concept of self-selection and a response to questions of participants regarding the usage of the injured or uninjured hand for the described tasks.

Another frequent comment was that several items were too masculine. This was also described by Konzelmann et al. [18], who stated that the tasks depicted in items 53–62 are heavy activities more specific to men. Overall, in the development of the HFS, the authors tried to balance gender [10]. Adjusting the HFS to make it less masculine would indicate more rigorous changes in the tasks and therefore the construct.

The HFS is a questionnaire developed in the early 1990s, using pictures from that era. In the past 25 years, some activities and tools have changed, for example, the use of a rotary opener and cash money is less common. The pictures should be updated to match the current time frame.

For testing the prefinal version of the HFS-DLV, part of the participants had a diagnosis not classified as specific or nonspecific CANS. We assumed this would not affect the comments on the comprehensibility of the items. To prevent bias, none of the participants contributing to part 1 of the study were involved in the analysis for the psychometric properties of the final HFS-DLV, although we did not change any of the items.

### Part 2: measurement properties of the HFS-DLV

In total, 6 out of 12 (50%) predefined hypotheses were accepted, which was below the goal of 75%. The highest correlation was found between the HFS-DLV and the QuickDASH, which is in line with the high correlation between HFS-F and the DASH [18]. The HFS-DLV was also strongly correlated to the PRWHE, which might be explained by the finding that the PRWHE and DASH strongly correlate due to the assessment of comparable constructs [42].

Our hypotheses for the correlations between HFS-DLV and NRS pain, RAND-36 vitality, and RAND-36 mental health could not be accepted. For all three, a slightly higher correlation then predicted was found.

For the NRS pain, a weak to moderate correlation was predicted, but a strong correlation was found. The predefined hypothesis was based on previous literature and a recent study who found a weak correlation between the HFS and VAS pain (coefficient of − 0.247) [18]. The average score on the NRS pain was similar with 4.6 vs. 4.9 to Konzelmann [18]. On the other hand, the pathology underlying the pain was different, in the study of Konzelmann [18]; more than half of the participants had shoulder pathology, and only one third had hand/wrist pathology. For all items in the HFS, an individual needs the functionality of the hands and wrists; only a small portion of items require intensive use of the shoulders. This might explain why patients with pain from hand/wrist disorders show a stronger correlation with the HFS.

Our assumed correlation for the HFS-DLV with the RAND-36 vitality was weak-moderate, but we found a strong correlation, although this finding was marginally higher than expected. It might be that participants who experience more fatigue and who have less energy, experience more troubles performing the tasks in the HFS-DLV than predicted. For the RAND-36 mental health, a weak correlation was assumed, but a moderate correlation was found. Based on the biopsychosocial model [43], it can be argued that not only hand/wrist function but also psychological well-being plays an important role for a person when determining his or her ability to perform a specific task. Konzelmann et al. [18] found a weak correlation with the SF-36 mental component summary, however their sample consisted almost completely of men (84%) and this might play a role in the observed difference.

All three hypotheses for known-groups validity were correct but not of statistically significant difference, although the employment state showed a trend toward significance. For the employment state, only participants with a paid job were included. Participants with voluntary employment and students were categorized as unemployed. This could have affected the outcome, since these participants potentially could be able to perform a paid job. Nearly half of the participants had complaints of both hands, which meant the dominant side was in almost all cases affected. It was, however, not known whether one hand was more affected than the other. Considering the relatively small number of participants, a significant difference might be hard to determine.

Since there was no gold standard to determine the validity of the HFS-DLV, using predefined hypotheses for construct validity seems eligible. Possibly the hypotheses were too strict, since the three hypotheses that were incorrect only slightly differed from the predicted correlations. Alternatively, the validity could be assessed by comparing the HFS-DLV to more objective manners to determine work capacity, such as the Functional Capacity Evaluation (FCE) testing, as has also been performed previously for the English version of the HFS by Matheson et al. [10]

The internal consistency of the HFS-DLV appeared to be higher than deemed acceptable. Although the recommended total of 434 participants was not reached, with 119 participants an adequate interpretation could be made. A remarkable finding was the very high Cronbach’s alpha (0.98), which tends to be higher when a questionnaire has more items, suggesting redundancy. A similarly high internal consistency has been described before [18]. Since the HFS has 62 items, redundancy might indeed be present. A high number of items can lead to less motivation toward the end of the questionnaire, especially when all the questions have the same outline and instructions. Furthermore, for a quick evaluation of a person’s functioning in clinical practice, less items are preferable. In further research, the assumed redundancy of the HFS-DLV should be investigated, for example, using factor analysis.

The test-retest reliability determined by the ICC was good and appeared to be comparable with previous research [18]. The Bland-Altman method showed a centered distribution, with limits of agreement slightly higher than those found by Konzelmann et al., who used a smaller interval (48 h instead of up to 3 weeks) between the two administrations of the HFS [18]. However, even though we did not actually assess whether or not change in the clinical situation occurred, we did not expect these patients to improve or deteriorate considerably within this interval because of their generally long-standing complaints and absence of treatment during this interval. Since it has a low degree of measurement error, this implies that the HFS-DLV can be used for repeated measures in clinical practice. We determined the measurement properties in a group of patients with CANS from an outpatient hospital and from peripheral hand therapy practices. The test-retest reliability of the original HFS was tested in 48 patients with various upper extremity impairments, including hand fractures, carpal tunnel syndrome, and lacerations [10]. Konzelmann et al. [18] investigated a population of hospitalized patients admitted for rehabilitation with upper limb complaints. In all these populations with various upper extremity diseases, the HFS was found to have reasonable to good test-retest reliability.

Responsiveness determined by the AUC was good, although the SDC and MIC were quite high (45/248 and 37/248, respectively). Our SEM of 16.2 is similar to that found by Benhissen et al., but the MIC reported by them is lower (26/248) [44]. This might be explained by a different method to determine the ROC cut-off point or actual differences in MIC, e.g. due to differences in patient characteristics. Although the HFS is able to discriminate between subjects who have and who have not improved, an improvement in score between 37 and 45 points should be interpreted with caution [33]. A good responsiveness is clinically important to be able to use the HFS-DLV in daily practice or research to evaluate treatment effects, an important objective of PROs in general.

We observed that some participants filled in more than six question marks on the HFS-DLV, indicating that the questionnaires were marginally reliable. A question mark gives a similar score as if a person is unable to do the task. This could have given an underestimation of the participants’ abilities. Answering with a question mark was not observed in testing the prefinal version of the HFS-DLV. It seemed to make a difference if a researcher was present or not. In the additional comments of the HFS-DLV, participants explained that they chose a question mark when they had never done the tasks stated in the questionnaire. In the current HFS participant instructions, it is not stated what a participant should fill in when they have never done the task before. The general procedure for administration of the HFS states that under guidance of an evaluator, the participant should complete the first two items of the questionnaire. If the evaluator is assured that the participant understands the instructions adequately, the participant can complete the remaining items independently. However, the first two items are frequently encountered tasks with which all participants are familiar. A statement that participants should make a good guess in case of tasks they never performed before could be a valuable addition to the instructions. It would be more practical and less time consuming if a participant could complete the HFS-DLV without the presence of an evaluator. Another possibility would be to exclude the option of the question mark, which would force people to make a choice, but this could lead to incomplete questionnaires. Unreliable questionnaires (≥4 points difference between the similar items of internal check) were more observed for the test-retest reliability and responsiveness analyses. This can be explained by the fact that participants had to complete the HFS-DLV twice. This observation is also an argument to try to reduce the number of items on the HFS.

The strength of this study was the adherence to COSMIN recommendations to assess measurement properties, in particular the use of a wide variety of 6 questionnaires to determine construct validity.

The limitations of this study include the high number of marginally reliable questionnaires, which could possibly be reduced if a researcher would be present at completion of the questionnaires. We investigated patients with specific and nonspecific CANS in our study, so the presented results could possibly be less applicable to patients with hand/wrist pathology caused by trauma and/or systemic disease. Furthermore, the various measurement properties were not all assessed in the same sample, but generally in either a UH or PHTP group. While the majority of patient characteristics was similar, the distribution of diagnoses differed, which might limit generalization of the results. If that were the case this would probably hold true more for construct validity and responsiveness than for internal consistency and test-retest reliability. Further research might focus on determining or confirming the measurement properties of the HFS-DLV in other groups of patients.

## Conclusions

The 5-step translation process and adaptation of the HFS into the HFS-DLV went according to plan, although some items were difficult to translate into Dutch. For the construct validity of the HFS-DLV, the presumed direction of the correlations was correct, but less than 75% of the hypotheses were confirmed. Internal consistency was high, suggesting redundancy. The test-retest reliability and responsiveness of the HFS-DLV were good. No floor or ceiling effects were found. Therefore, the HFS-DLV can be used in research and clinical practice for Dutch patients with CANS, e.g., to evaluate self-reported functional work ability.

Cross cultural translation and adaptation of the HFS can also be useful for other languages than English, French, or Dutch, but we recommend investigating item reduction and updating the items to the current time frame before putting more effort into additional translations.

## Data Availability

The datasets used and/or analyzed during the current study are available from the corresponding author on reasonable request.

## Abbreviations

AUC: Area under the ROC curve
CANS: Complaints of arm, neck, and shoulder
DASH: Disabilities of the Arm, Shoulder, and Hand outcome measure
FCE: Functional Capacity Evaluation
GRS: Global rating scale
HFS: Hand Function Sort
HFS-DLV: HFS-Dutch Language Version
ICC: Intraclass correlation coefficient
IQR: Interquartile range
LoA: Limits of agreement
MIC: Minimal important change
NRS-pain: Numeric Pain Rating
PDC: Physical demand characteristics
PDI: Pain Disability Index
PRO: Patient-reported outcome
PRWHE: Patient-Rated Wrist/Hand Evaluation
RPC: Rating of perceived capacity
SDC: Smallest detectable change
SEM: Standard error of measurement
WAS: Work Ability Score
